# The Correlation of Acne with Anxiety after Rhinoplasty

**Published:** 2019-05

**Authors:** Hossein Kavoussi, Ali Ebrahimi, Mansour Rezaei, Habibolah Khazaie, Jalal Shakeri, Sajedeh Jamshidi, Reza Kavoussi

**Affiliations:** 1 *Department of Dermatology, Hajdaie Dermatolgy Clinic, Kermanshah University of Medical Sciences* *, Kermanshah, Iran.*; 2 *Family Health Research Center, Health School, Kermanshah University of Medical Sciences, Kermanshah, Iran. *; 3 *Sleep Disorders Research Center, Kermanshah University of Medical Sciences, Kermanshah, Iran.*; 4 *Farabi Hospital, Kermanshah University of Medical Sciences, Kermanshah, Iran.*; 5 *Students Research Committee, Kermanshah University of Medical Sciences, Kermanshah, Iran.*

**Keywords:** Anxiety, Acne, Rhinoplasty, State-trait anxiety

## Abstract

**Introduction:**

Rhinoplasty is one of the most common cosmetic surgeries occasionally associated with complications, such as acne lesions. Anxiety reportedly leads to the emergence or exacerbation of acne lesions.

**Materials and Methods:**

This cross-sectional study was conducted on 147 patients undergoing rhinoplasty. The patients were assigned into two groups of case (with acne lesions) and control (without acne lesions) entailing 52 (45 females, 7 males) and 95 (68 females, 27 males) subjects, respectively. The data were collected using an instrument entailing clinical and demographic data and the state-trait anxiety inventory developed by Spielberger.

**Results:**

According to the results, 70.7% and 71.6% of the patients in the case and control groups were female, respectively. Regarding the education level, 61.5% and 68.4% of the case and control groups had academic education, respectively. Additionally, 61.5% and 42.1% of the subjects in the case and control groups were single with the mean ages of 25.06±5.077 and 27.45±5.909 years, respectively. , 73.1% of the case group indicated grade 2 acne mostly in the face (100%) appearing 1-4 weeks post-surgery (51.9%). The case group had higher mean scores of trait (46.92±12.53) and state (46.21±9.30) anxiety, trait (P=0.001) and state (P=0.019) anxiety severity, as well as the prevalence of cosmetic dissatisfaction (51.9%), compared to the control group. Furthermore, acne severity showed a direct correlation with the severity of trait anxiety (r=0.472, P=0.005) and state anxiety (r=0.443, P=0.013).

**Conclusion:**

As the findings indicated, anxiety could be a major factor in triggering or exacerbating acne lesions after rhinoplasty. The assessment of mental health before the surgery, proper selection of the cases seeking aesthetic surgery, provision of psychological support, lack of medical history of acne lesions, and successful cosmetic rhinoplasty could result in reducing the prevalence of acne lesions after cosmetic surgeries, especially rhinoplasty.

## Introduction

Rhinoplasty is one of the most frequent and interesting cosmetic surgical procedures in the world, targeted toward the improvement of facial appearance (1,2). There has been an increasing tendency toward this aesthetic procedure among teenage girls and young adults, especially in Iran. However, the surgeon should be aware of the motivation and expectations of the patients and inform them about its associated complications prior to the surgery (3).Acne is a common skin disorder resulting from four factors, including seborrhea, colonization of *Propionibacterium acne,*hypercornification of the pilosebaceous duct, and inflammation (4,5). Many risk factors, such as mental disorders, diet, drug consumption, and use of cosmetics, account for triggering or exacerbating acne lesions (5-10). The most common neuropsychiatric disorders in the patients with acne vulgaris include anxiety, depression, anger, reduced self-esteem, suicidal thoughts, and problems with self-image and interpersonal relationships (11-13). The majority of the studies have reported a positive correlation between acne and anxiety (5-7,11-13). However, a number of studies have indicated no correlation between these two variables (14,15). Based on the evidence, most of the patients undergoing rhinoplasty experience the emergence or exacerbation of acne lesions (2,16-18). As mentioned earlier, there is a growing tendency toward rhinoplasty, which is reportedly followed by an incidence of acne lesions. Regarding this and considering the role of anxiety in the pathogenesis of acne lesions, the present study was conducted to assess the level of anxiety in the patients suffering from acne lesions after undergoing rhinoplasty.

## Materials and Methods

Study Design 

This cross-sectional study was conducted on 147 cases having undergone rhinoplasty in the hospitals of Kermanshah city, Iran, and referring to Hajdaei Dermatology Clinic in Kermanshah city over 5 years (2009-2014). For the purpose of the study, 52 patients suffering from the emergence of acne lesions or the exacerbation of this condition after rhinoplasty were assigned into the case group. On the other hand, 95 cases having minimal skin problems without any acne lesions were considered as the control group. In the case group, the acne lesions were documented by at least two dermatologists. The onset or exacerbation of acne lesions after the surgery in the case group occurred in less than 3 months. The exclusion criteria were: 1) use of comedogenic cosmetic products, 2) use of drugs inducing acne, 3) affliction with post-epilation folliculitis, and 4) development of acne lesions only under adhesive tape.

*Data Collection*

The data were collected using an instrument consisting of three parts. The first section covered the patients’ demographic characteristics. In the second section, the clinical characteristics of acne lesions in the case group were recorded based on the clinical findings. In the third section, the anxiety status of the two groups was evaluated by the State and Trait Anxiety Inventory (STAI) developed by Spielberger.

The patient’s level of satisfaction with rhinoplasty procedure in both groups was rated as low, moderate, and high. Acne severity was determined using a grading system used by an Indian author (19) categorizing acne lesions into four grades. This classification included: 

Grade 1: Comedones and occasional papules

Grade 2: Papules, comedones, and few pustules

Grade 3: Predominant pustules, nodules, and abscesses.

Grade 4: Mainly cysts, abscesses, and widespread scarring

The determination of state-trait anxiety was accomplished using the self-rating STAI, including two equal parts. Each part consisted of 20 items rated on a 4-point Likert scale (ranging from 1 to 4). 

Accordingly, the minimum and maximum scores of this inventory are 20 and 80, respectively. In this instrument, the scores of 20-39, 40-59, and 60 ≤ represent mild, moderate, and severe anxiety levels, respectively. The validity and reliability of the Persian version of the STAI have been documented for different populations in Iran in multiple studies. In the measurement of the co nstruct validity of this instrument through factor analysis, the Kaiser Meyer Olkin (KMO) measure of sampling adequacy (KMO=0.824 0.905) was indicative of thatacceptable inter-item correlations (P=0.001). The Cronbach’s alpha coefficient for the state and trait anxiety scales were obtained as 0.70 and 0.78, respectively (20,21).

*Ethical Considerations*

This study was approved by the Ethics Committee of Kermanshah University of Medical Science, Kermanshah, Iran. In line with the ethical principles of research, the information of all participants was kept confidential. Furthermore, written informed consent was obtained after explaining the research objectives to all subjects.

*Statistical Analysis*

Chi-square test was used to compare anxiety disorder in both groups. Age and gender were matched by independent- sample t-test and Chi-square test, respectively. Analysis of qualitative data was performed by Chi-square and Fisher's exact tests. Furthermore, the correlation between the severity of anxiety and acne was determined using the Chi-square test and contingency coefficient (r). P-value less than 0.05 was considered statistically significant. All data analyses were performed in SPSS software (version 16).

## Results

The case group consisted of 45 (70.7%) females and 7 (29.3%) males with the mean age of 25.06±5.077 years (age range: 18-45 years). Furthermore, the control group included 68 (71.6%) females and 27 (28.4%) males with the mean age of 27.45±5.909 years (age range: 20-44 years; Table.1). The results indicated no significant difference between the two groups in terms of gender, education level, age, and marital status (Table.1).

**Table 1:** Demographic data and cosmetic outcomes in the case and control groups

**Variables **	**Case group (n= 52)**	**Control group (n=95) **	**P-value**
age (mean) years	25.06±5.077	27.45±5.909	0.011
Gender			
Female n (%)	45 (70.7%)	68(71.6%)	0.2
Male n (%)	7 (29.3%)	27 (28.4%)	
Level of education			
Under diploma n (%)	7 (13.5%)	12 (12.6%)	
Diploma n (%)	13 (25%)	18 (18.9%)	0.212
Academic education n (%)	32 (61.5%)	65 (68.4%)	
Marital status			
Married n (%)	10 (19.2%)	33 (34.7%)	
Single no (%)	32 (61.5%)	40 (42.1%)	0.83
Divorced n (%)	10 (19.2%)	22 (23.1%)	
Cosmetic satisfaction			
Low n (%)	27 (51.9%),	10 (10.5%)	
Moderate no (%)	17 (32.7%)	47 (49.5%)	0.001
High n (%)	8 (15.4%)	38 (40%)	

With regard to the satisfaction level with the cosmetic rhinoplasty, 27 (51.9%), 17 (32.7%), and 8 (15.4%) patients in the case group had low, moderate, and high satisfaction levels, respectively. In terms of the control group, cosmetic satisfaction was rated as low, moderate, and high in 10 (10.5%), 47 (49.5%), and 38 (40%) subjects, respectively. Cosmetic satisfaction was higher in the control group than in the case group (P=0.001; Table.1). In the case group, 38 (73.1%), 12 (23.1%), and 2 (3.8%) acne lesions had severity grades 2,3 and 4, respectively. Acne lesions were located in the face in 48 (92.3%) patients; furthermore, in 4 (7.7%) patients, they appeared both in the face and trunk concurrently. The onset of postrhinoplasty acne occurred in 14 (26.9%), 27 (51.9% ), and 11 (21.2%) patients in less than one week, more than one week and less than four weeks and more than four weeks, respectively. Furthermore, 31 (59.6%) patients had a previous history of mild acne lesions exacerbated after this surgery, while 21(40.4%) patients had no previous history of acne lesion (Table.2).

**Table 2:** Clinical characteristics of acne lesions in the case group

**Variables **	**n (%)**
Severity of acne lesions	
Grade 1	0 (%)
Grade 2	38 (73.1%)
Grade 3	12 (23.1%)
Grade 4	2 (3.8%)
Time of onset	
≥1 weeks	14 (26.9%)
≤1 week and ≥4 weeks	27 (51.9%)
≤4 weeks	11 (21.2%)
Locations	
Face	48 (92.3%)
Face plus trunk	4 (7.7%)
Previous history of acne	
Yes	31 (59.6%)
No	21 (40.4%)

Regarding the trait anxiety, in the case group, 14 (26.9%), 26 (50%), and 12 (23.1%) cases showed mild, moderate, and severe trait anxiety levels, respectively. In the control group, 46 (48.4%), 43 (45.3%), and 6 (6.3%) individuals showed mild, moderate, and severe trait anxiety levels, respectively. 

The results of the Pearson’s Chi-square test revealed that the severity of trait anxiety was significantly higher in the case group, compared to that in the control group (χ2=17.779, P=0.001; Table.3).

**Table 3:** Characteristics of state-trait anxiety in the case and control groups

**Variables**	**Case group(n=52)**	**Control group(n=95)**	**P-value**	**Test statistics**
**Severity of trait anxiety**				
Mild	14 (26.9%)	46 (48.4%),		Chi-square
Moderate	26 (50%)	43 (45.3%)	0.001	17.779
Severe	12 (23.1%)	6 (6.3%)		
**Severity of state anxiety**				
Mild	10 (19.2%)	42 (44.2%)		Chi-square
** Moderate**	31 (59.6%)	48 (50.5%)	0.019	11.815
Severe	11 (21.2%)	5 (5.3%)		
**Mean score of trait anxiety**	46.92±12.53	40.07±9.30	0.001	t-test=3.762
Mean score of state anxiety	46.21±9.30	40.22±9.48	0.001	t-test=3.494

In terms of the state anxiety, 10 (19.2%), 31 (59.6%), and 11 (21.2%) patients in the case group showed mild, moderate, and severe levels of anxiety, respectively. In the control group, 42 (44.2%), 48 (50.5%), and 5 (5.3%) individuals had mild, moderate, and severe state anxiety levels, respectively. The severity of state anxiety was significantly different between the two groups (χ^2^=11.815, P=0.019; Table.3).

The mean scores of trait anxiety were 46.92±12.53 and 40.07±9.30 in the case and control groups, respectively. Furthermore, the mean scores of state anxiety were obtained as 46.21±10.72 and 40.22±9.48 in the case and control groups, respectively. There was a significant difference between the case and control groups in terms of the mean scores of state and trait anxiety (P<0.05; Table 3). However, acne severity showed no direct correlation with the severity of trait (r= 0.472, P=0.005) and state anxiety (r= 0.443, P=0.013; Table.4).

**Table 4:** Correlation between the severity of anxiety and acne

		**Acne severity**			**Total**	**r and P-value**
		Grade 2	Grade 3	Grade 4		
State anxiety severity	Mild n (%)	9 (23.7%)	1 (8.3%)	0 (0.0%)	10 (19.2%)	r=0.443 P=0.013
	Moderate n (%)	26 (68.4%)	5 (41.7%)	0 (0.0%)	31 (59.6%)	
	Severe n (%)	3 (7.9%)	6 (50.0%)	2 (100.0%)	11 (21.2%)	
Trait anxiety severity	Mild n (%) Moderate n (%) Severe n (%)	12 (31.6%) 22 (57.9%) 4 (10.5%)	2 (16.7%) 4 (33.3%) 6 (50.0%)	0 (0.0%) 0 (0.0%) 2 (100.0%)	14 (26.9%) 26 (50.0%) 12 (23.1%)	r=0.472 P=0.005

r=contingency coefficient

## Discussion

As the findings of the present study indicated, the patients suffering from acne had significantly higher state-trait anxiety severity and mean score after undergoing rhinoplasty than the control group (P<0.05). Additionally, 73.1% of the case group had grade 2 acne mostly in the face appearing 1-4 weeks postsurgery. The results revealed that the severity of acne lesions was higher in the patients with poor cosmetic outcomes and severe state-trait anxiety.

Consistent with our findings, in the majority of the studies (2,17), mostly females and patients with 30-40 years of age have been reported to suffer from acne after rhinoplasty. Belli et al. found significant differences between the case and control groups regarding anxiety in the patients seeking rhinoplasty (22). However, Ercolani et al. found a significant decrease in anxiety in the patients following rhinoplasty (23). The present study is the first attempt assessing anxiety in the postrhinoplasty patients suffering from acne lesions.

In many studies, the prevalence of anxiety in the patients suffering from acne lesions was higher than that in controls (5-7,11-14,24). A number of studies have reported a correlation between the severity of anxiety and severity of acne (25-27). In the current study, the patients suffering from acne had a higher prevalence of anxiety. Furthermore, there was a direct correlation between the severity of anxiety and acne in this group.

Nemati et al. found that 42.9% of the cases developed mild acne after rhinoplasty (2), and 27% of them showed acne exacerbation over a month postoperation. However, most of our patients presented moderate or grade 2 acne. In addition, the onset of acne lesions occurred between the first and fourth weeks after rhinoplasty in half of the patients. Almost 60% of our patients with a history of acne lesions showed the recurrence and exacerbation of acne after rhionoplasty. Therefore, the past medical history of acne is the main risk factor for the development and exacerbation of acne after rhionoplasty.

In the present study, only 7.7% of the patients showed acne lesions on both face and trunk concurrently. Choi et al. concluded that the location of acne lesions was significantly affected by sebum level. Furthermore (28), Zouboulis stated that rhinoplasty induces stress to the skin, thereby resulting in the release of corticotrophin-releasing hormones from sebocytes that finally increase the sebeum secretion of sebaceous glands (29). These reasons could justify the increased prevalence of acne after rhinoplasty, especially on the face. 

In the current study, the case group had worse cosmetic outcomes; moreover, the severity of acne was more prevalent in the patients with unpleasant cosmetic outcomes. These findings are consistent with those obtained by Coban stating that the cosmetic success of rhinoplasty was accompanied with the reduced severity and symptoms of acne lesions (27).

## Conclusion

Based on the findings of the current study, it can be concluded that anxiety is an important factor in the exacerbation or emergence of acne lesions in patients after undergoing rhinoplasty. Therefore, the assessment of mental health before the surgery, proper selection of the cases seeking aesthetic surgery, provision of psychological support, treatment, and evaluation, lack of medical history of acne lesions, and successful cosmetic rhinoplasty could result in reducing the prevalence of acne lesions after cosmetic surgeries, especially rhinoplasty. The limitations of the present study included the non-evaluation of the neuropsychiatric status of the patients before the surgery, small sample size, implementation of a monocenter study, and evaluation of the cosmetic outcomes of rhinoplasty only from the patients’ perspectives.
